# Overcoming recruitment barriers revealed high readiness to participate and low dropout rate among people with schizophrenia in a randomized controlled trial testing the effect of a Guided Self-Determination intervention

**DOI:** 10.1186/1471-244X-14-28

**Published:** 2014-02-03

**Authors:** Rikke Jørgensen, Povl Munk-Jørgensen, Paul H Lysaker, Kelly D Buck, Lars Hansson, Vibeke Zoffmann

**Affiliations:** 1 Unit for Psychiatric Research, Aalborg Psychiatric Hospital, Aalborg University Hospital, Mølleparkvej 10, 9000 Aalborg, Denmark; 2 Department M, Aarhus University Hospital, Aarhus, Denmark; 3 Roudebush Veteran Affairs Medical Center, Indianapolis, IN, USA; 4 Indiana University School of Medicine, Indianapolis, USA; 5 Lund University, Lund, Sweden; 6 Steno Diabetes Center, Gentofte, Denmark; 7 NKLMS Oslo University Hospital, Oslo, Norway

**Keywords:** Schizophrenia, Outpatients, Randomized controlled trial, Organization, Recruitment challenges, Strategies, Readiness

## Abstract

**Background:**

Recruitment is one of the most serious challenges in performing randomized controlled trials. Often clinical trials with participants diagnosed with schizophrenia are terminated prematurely because of recruitment challenges resulting in a considerable waste of resources in the form of time, funding, and the participants’ efforts. Dropout rates in schizophrenia trials are also high.

Recruitment challenges are often due to patients not wanting to participate in research but can also be due to clinicians’ concerns regarding individuals diagnosed with schizophrenia as participants in research. This paper reports how overcoming recruitment challenges not related to patients revealed high readiness to take part and low dropout rates in a one year long randomized controlled trial testing Guided Self-Determination (GSD) among outpatients with schizophrenia receiving treatment in Assertive Outreach Teams in the northern part of Denmark.

**Methods:**

GSD is a shared decision-making and mutual problem-solving method using reflection sheets, which was developed in diabetes care and adjusted for this study and utilized by patients with schizophrenia. Descriptive data on strategies to overcome recruitment challenges were derived from notes and observations made during the randomized controlled trial testing of GSD in six outpatient teams.

**Results:**

Three types of recruitment challenges not related to patients were identified and met during the trial: 1) organizational challenges, 2) challenges with finding eligible participants and 3) challenges with having professionals invite patients to participate. These challenges were overcome through: 1) extension of time, 2) expansion of the clinical recruitment area and 3) encouragement of professionals to invite patients to the study. Through overcoming these challenges, we identified a remarkably high patient-readiness to take part (101 of 120 asked accepted) and a low dropout rate (8%).

**Conclusion:**

Distinction between recruitment challenges was important in discovering the readiness among patients with schizophrenia to take part in and complete a trial with the GSD-intervention.

## Background

Randomized controlled trials (RCTs) are widely accepted as the most powerful design for evaluating the efficacy and effectiveness of health care interventions. However, challenges with recruitment in RCTs are known to be common and complex in health care, and frequently more difficult than anticipated [1-4]. In a review from 2002, Gilbody et al. reported that in the past 50 years less than 5% of schizophrenia trials had reached sufficient power [5]. Further, it is a common understanding that especially people with schizophrenia are difficult to recruit into trials [5-7]. Additionally, dropout rates in drug trials with people with schizophrenia were reported to be 65.9% with the implication that dropout rates increase by length of trial duration [8]. Also withdrawal from the treatment without withdrawal from the trial is a common challenge. In a review of 74 trials testing psychosocial treatment among individuals diagnosed with schizophrenia, withdrawal either prior to or during treatment was estimated to be approximately 13% [9]. Delays in trials, withdrawal from treatment in trials, high dropout and underpowered trials have consequences with increases in cost and shortcomings in the scientific value of the intervention being tested [10].

As a variety of challenges to recruitment and completion of trials can be at play, distinctions between these challenges and barriers have been attempted and investigated separately. Clinicians’ attitudes about recruiting participants with severe mental illness for trials have been investigated in qualitative studies [2,11]. Among other things, clinicians were less likely to refer patients diagnosed with schizophrenia if they lacked knowledge of research methods and trials, had concerns of increased workload or gave research a low priority. Some clinicians were also likely to decide their own inclusion criteria and make independent decisions on behalf of their patients. These attitudes of recruiting clinicians might be related to the fact that research participation of patients diagnosed with schizophrenia is an issue of concern stated both by clinicians and researchers [12]. Concerns are leveled at patients’ decisional capacity [13], understanding of ethical considerations [14], and nature of voluntarism and motivations for participating in research [15].

Among individuals with schizophrenia, recruitment challenges and barriers as well as the above reasons for participating in research have been explored. Challenges and barriers regarding participation in research have been connected with timing of approach, practical barriers, conceptualization of mental health problems, concerns of potential harm, and negative influence of other patients [16,17]. Regarding reasons for participating in research Chong et al. [15] found two main reasons for volunteering; 1) the possibility that I might get well, 2) I’m helping other patients like myself, and a similar study Roberts et al. [18] found three main reasons; 1) helping other people, 2) helping science, 3) gaining a sense of hope.

In order to further investigate the perspective of the potential participant, patients with severe mental illnesses admitted to a psychiatric hospital were interviewed about their potential interest and readiness to participate in research. Patients diagnosed with schizophrenia were generally more reluctant to participate in trials, less convinced that they would benefit from a new treatment and indicated less often “to help science and other patients” as their reason to participate compared to patients with other mental disorders [19].

Within trial research it seems that the biggest challenge is recruitment of potential participants.

Researchers must expect challenges regarding recruitment due to patients not wanting to participate or clinicians’ concerns regarding individuals diagnosed with schizophrenia as participants in research. Also factors such as low readiness to participate, withdrawal from treatment and high dropout must be anticipated.

As recommended by Halpern et al. [10], we attempted to identify potential recruitment challenges in our trial beforehand and designed the trial according to those considerations. Despite these considerations we nevertheless experienced challenges with recruitment.

This paper reports how recruitment challenges not related to patient issues were overcome and revealed the true readiness to take part in and complete a complex clinical trial testing Guided Self-Determination (GSD) among outpatients with schizophrenia in the northern part of Denmark.

## Method

### Ethics

The trial was approved by the Danish Data Protection Agency and The Danish National Committee on Biomedical Research Ethics under number VN-20070070. The trial was also registered at ClinicalTrials.gov with identifier: NCT01282307.

### Design

The study design was descriptive, and based on data, notes, and observations from a randomized controlled trial testing a novel intervention in six outpatient teams.

During the recruitment period all eligible participants were registered in a logbook containing information about: participation in information meetings, reasons for declining or participating in the trial, and comments regarding the trial from either the eligible participant or his/her mental health care professional (MHCP).

The observations and notes from the recruitment period and the data collection were used to describe and distinguish the recruitment challenges not related to patients from those connected with patients’ readiness to take part in and complete the trial. Further, the strategies chosen to overcome them were described. In addition, data from the trial were registered to report and calculate proportions of withdrawal from treatment and dropout from the trial. Withdrawal from treatment occurs when participants withdraw from the intervention being tested in the trial either prior to or during the treatment period but continue in the data collection in the trial. Dropout is defined as the participants who left the trial before the trial end [8,9].

### The RCT

A total of 101 outpatients from three Assertive Outreach Teams (AOT) and three District Teams (DT) in the northern part of Denmark participated in the study. Fifty-one were randomly assigned to the intervention group which received an immediate individual training program consisting of approximately ten sessions with the Guided Self-Determination (GSD) method [20-24]. Fifty patients were randomly assigned to a waiting list for a 12-month delayed individual training program with GSD (control group). Both groups also continued treatment as usual during the entire trial. Participants completed four self-rating questionnaires, a demographic data sheet (containing data about medication, alcohol use, substance use and course of illness), an interview concerning psychopathology and an assessment of social functioning at baseline and after 3, 6, and 12 months conducted in their own home or in a team office according to the participants’ preferences. Thirty-six MHCPs, primarily registered nurses specially trained within psychiatry, provided the intervention and the principal investigator (PI) (first author) conducted the data collection.

### Setting

The study took place in Region North (0.6 million inhabitants), the smallest of Denmark’s five Regions. Psychiatric Health Care Services covers both inpatient (269 beds) and outpatient care (approximately 4700 patients yearly) and is publicly funded.

### Study population

To be eligible for the trial, participants had to receive treatment in an AOT or meet the requirements to receive treatment in an AOT. Requirements for patients in AOTs were: having frequent and/or long admissions poor or no improvement in psychopathology and illness management, frequent discontinuation of treatment, or relapse. Further inclusion criteria for the trial were: meeting the criteria for schizophrenia ICD-10 F.20.0-9 or schizoaffective disorder F.25.0-9 according to participants’ hospital records, age 18 – 70 years, ability to understand, speak and write Danish, and signed informed written consent. The AOT and DT treatment offered the following treatment modalities: psychopharmacology, cognitive behavioral therapy, cognitive milieu therapy, psycho-education, and social skills training, which was considered treatment as usual (TAU) during the trial. Exclusion criteria were: previous participation with the method GSD, having a mental handicap or diagnosis of dementia or an organic brain disease according to the hospital record, in need of an interpreter to understand, speak and write Danish, and withdrawal of informed consent. Statistical power calculation was performed which demonstrated that 50 participants in each group were needed.

### Study information site visits

Before recruitment of participants, all teams were visited by the PI and informed about the clinical trial and provided with education about how to conduct the intervention using the GSD method. Written information about the recruitment process, the clinical trial and the GSD method was given to each MHCP. Approximately 16 hours (over 2-3 days) of information and education were provided. During the recruitment period the PI visited, mailed and/or telephoned the teams and team members at least twice a month to discuss eligible participants and to encourage the MHCPs to participate in the trial, and was also available for questions and supervision during the entire trial, both in relation to the trial procedures and the intervention.

Information and education were given to all MHCPs (n = 43) in the six teams, as it was determined by the hospital management as mandatory to participate in the trial.

### Recruitment

The MHCPs completed the recruitment strategy as part of their clinical work. First, the MHCPs distributed a patient information sheet for all eligible participants and invited them to an individual appointment with the PI to further discuss the trial. When the eligible participants agreed to meet with the PI, the MHCP contacted the PI and made arrangements for a meeting. According to the person’s preferences the meetings took place either in the person’s own home or in a team office. At the information meeting the eligible participants often brought community support workers, spouses or their MHCP. During the meeting, detailed written information about the trial was provided, oral information was given, and the eligible participants were encouraged to ask questions. They were then given time to make the decision about participation. Some made their decision immediately and some made it after a few days. For those who signed the informed consent, an appointment for a baseline interview was made.

Recruitment was an ongoing process, which started with the implementation of the first AOT in February 2008 through July 2011.

### The guided self-determination method

GSD is a shared decision making and problem-solving method focusing on facilitating empowerment [25] developed from qualitative research using Grounded Theory [20,22,23] originally developed for Type 1 Diabetes. The method has been modified for other diagnoses, including schizophrenia [26].

The purpose of the method is to guide the individual through a process of exploring difficulties in living with an illness systematically and creatively by means of words and drawings and then to focus on which difficulties to change in order to develop internally motivated self-management of the illness [24]. GSD uses 21 reflection sheets designed to guide the participant and MHCP through a process of autonomy-supportive problem solving. The reflection sheets are completed by the participant before, and between conversations with their MHCP, with each entitled to have their own opinions and perceptions about the content. More details about the GSD method and a case study where an individual diagnosed with schizophrenia used GSD are elaborated on elsewhere [26].

The GSD training in the trial was conducted with the participants individually with an instructive and flexible program consisting of 10 sessions, each lasting 30-60 minutes. The intervention period was decided to be six months with a follow-up period of six months. No participants were excluded from the trial if they spent more than six months with the intervention.

## Results

**Figure 1:**
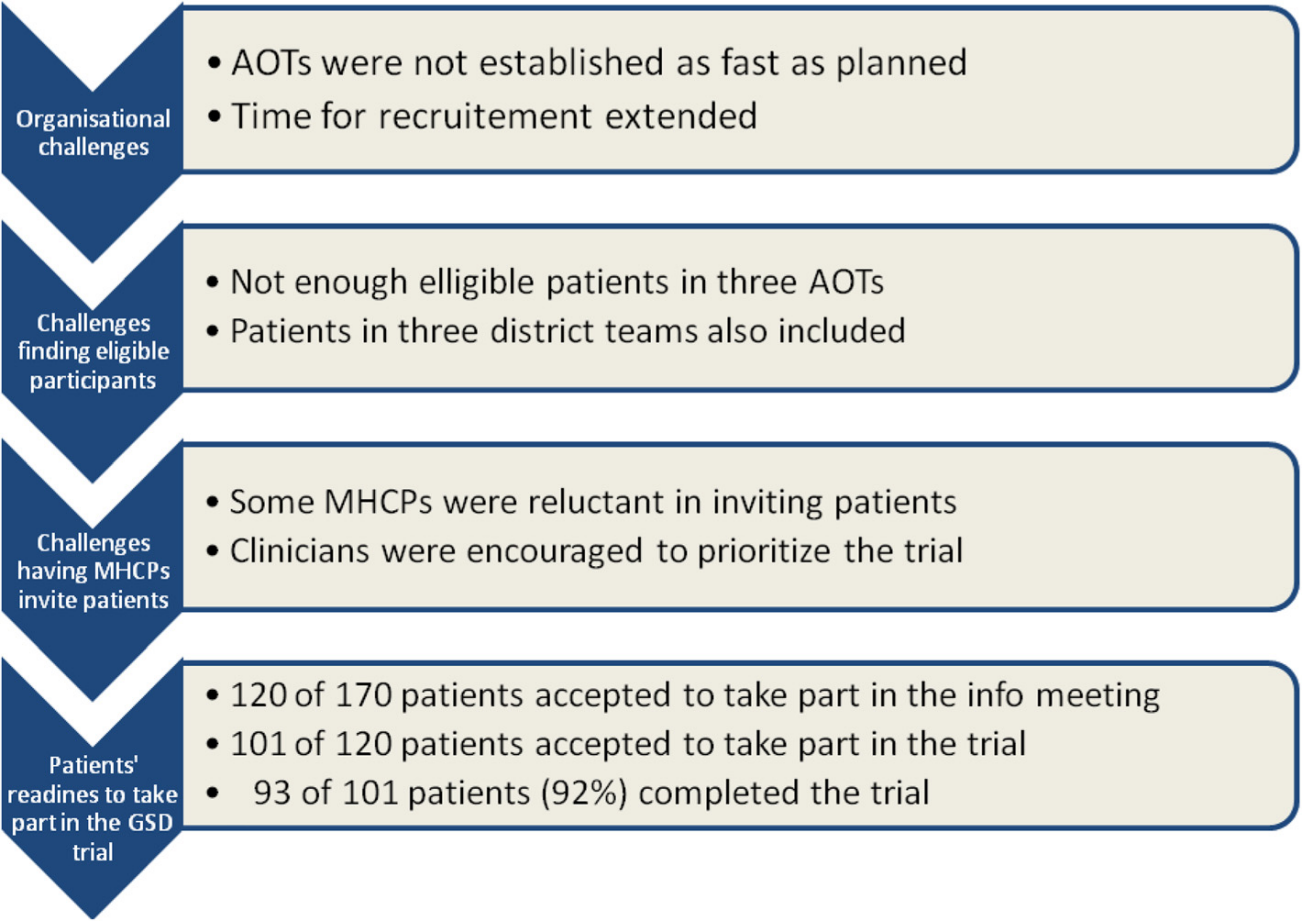
Overcoming recruitment challenges not related to patients revealed a high patient-readiness.

In the trial, barriers not related to patients were encountered. Three types of recruitment challenges not related to patients were identified: 1) organizational challenges, 2) challenges with finding eligible participants, and 3) challenges with having professionals invite patients. These challenges were overcome through: 1) extension of time, 2) expansion of the clinical recruitment area, and 3) encouragement of professionals to invite patients to the trial. Identifying and overcoming challenges not related to patients disclosed remarkably high patient-readiness to take part in the trial and a remarkably a low dropout rate (Figure 1: Overcoming recruitment challenges not related to patients revealed a high patient readiness).

### Challenges to recruitment not related to patients and strategies to overcome them

#### ***Organizational challenges***

Prior to the trial, the hospital management had decided that three future AOTs would be used for the study; therefore, the original trial protocol was designed in accordance with the plan of establishing the three AOTs and was considered to be feasible. Only a few months after the trial started, however, this plan was changed politically in order to reduce cost and because of difficulties in employing psychiatrists to work in the AOTs. This accounted for a considerable delay in the implementation plan. According to the original implementation plan, the three AOTs were supposed to be started in January 2008 to August 2008, but the last AOT was not implemented until August 2009, which ended up being a 12 month-delay.

**Figure 2:**
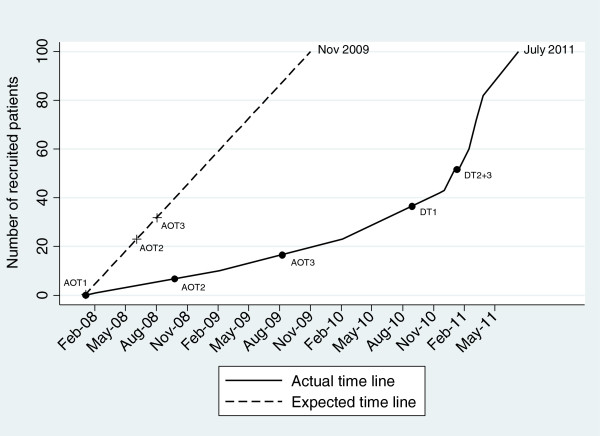
**Time line for recruitment in project GSD.** AOT1: Assertive Outreach Team1, AOT2: Assertive Outreach Team2, AOT3: Assertive Outreach Team3. DT1: District Team1, DT2: District Team2, DT3: District Team3.

To overcome this challenge and to avoid early termination of the trial and an underpowered trial, a time extension was judged necessary. The recruitment period was from January 2008 until November 2009, but was extended until July 2011 – an extension of 20 months (Figure 2: Time line for recruitment in project GSD).

#### ***Challenges finding eligible participants***

**Figure 3:**
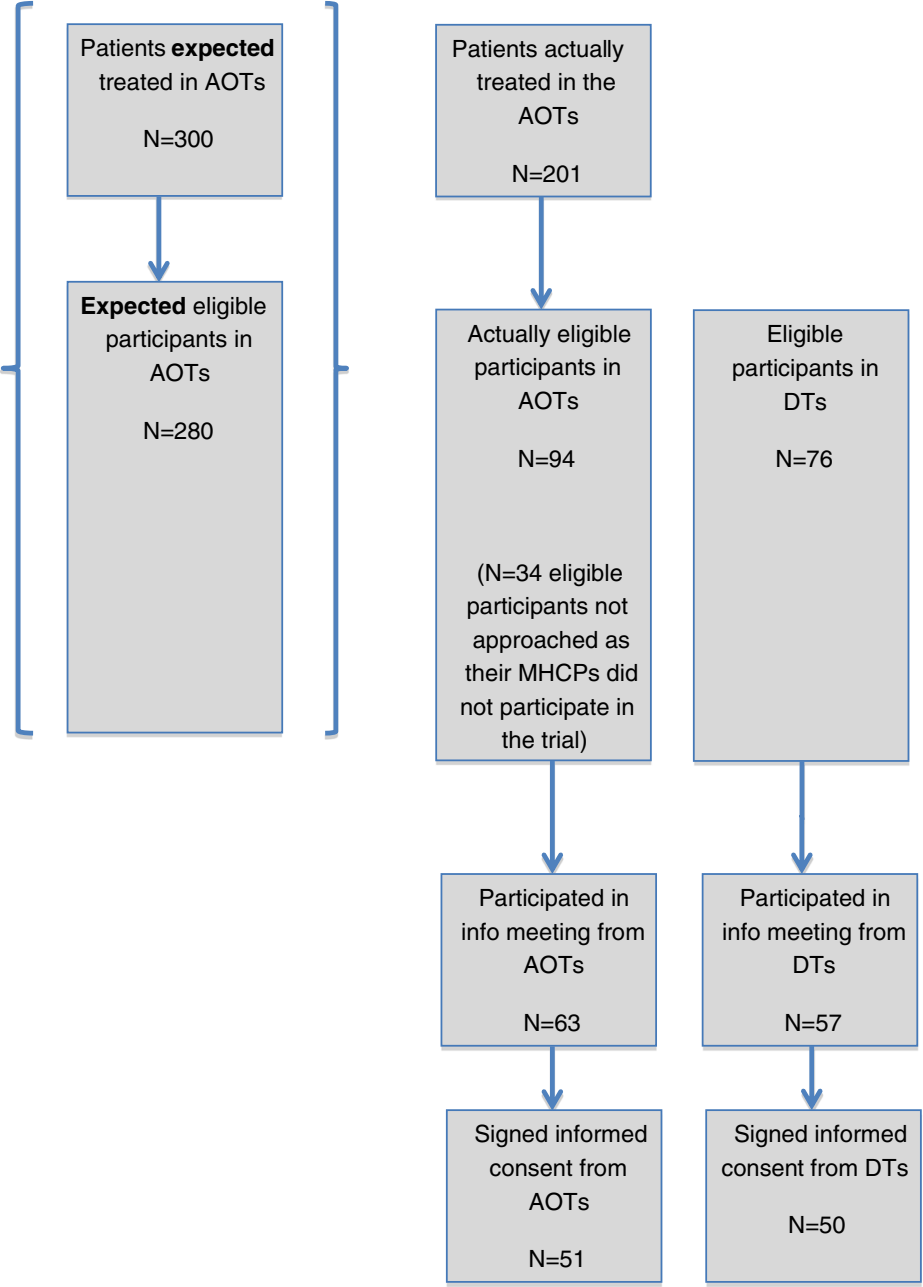
Flowchart for recruitment.

In addition to the delay in the implementation plan, the number of expected eligible participants for the trial decreased. The AOTs were expected to treat approximately 300 patients, and it was also expected that 90 to 95% of the patients (n = 280) in the AOTs would have a diagnosis of schizophrenia. In total, 24 MHCPs were expected to be employed, but due to changes in the implementation plan the actual number was 18 MHCPs, which reduced the total patients by a third. Some MHCPs resigned, and new ones were employed, but the total number was 18 MHCPs during the entire trial period. Without any proper explanation, the number of patients with a diagnosis of schizophrenia was smaller than expected. It appeared that only between 60 and 65% of the patients treated in the AOTs had schizophrenia, 30 to 35% less than expected. Looking at the numbers of patients in the three AOTs when the recruitment period ended, we found that 201 patients were treated in the AOTs and only 128 met the inclusion criteria for the trial. Five MHCPs from the AOTs did not participate in the trial; therefore 34 eligible participants were not approached or asked to participate in the trial. In total 94 eligible participants were present in the three AOTs compared to the expected 280 eligible participants in the implementation plan, a reduction of 66% (Figure 3: Flowchart for recruitment).

The challenge of finding enough eligible participants was overcome by an expansion of the clinical area. Three District Teams (DT) were included in the trial, and eligible participants from the DTs had to meet the requirements to receive treatment in an AOT besides the inclusion criteria for the trial. Seventy-six eligible participants were identified in the three DTs.

The MHCPs from the DTs had to undergo the same education as the MHCPs from the AOTs which also affected the time extension.

#### ***Challenges to having mental health care professionals invite patients***

When the MHCPs were informed and provided education about the trial, the recruitment process and the GSD method, they expressed several concerns. The concerns mainly consisted of how to manage the increase of workload and questions whether the eligible participants either were too severely ill to participate in research or to make the decision to participate in research.

In total, 20 MHCPs in the AOTs and 23 MHCPs in the DTs were informed and provided education about the trial. Altogether 36 of the 43 MHCPs took part in the trial as five MHCPs from the AOTs and two MHCPs from the DTs (in all 16%) did not participate.

Most of the non-participating MHCPs did not openly express their reluctance in the beginning, but communicated a kind of avoidance or “silent reluctance” by not answering mails or telephone calls from the PI, not attending meetings with the PI, or avoiding providing the information sheet to eligible participants. Two MHCPs actively denied informing eligible participants about the trial, as they believed that the trial would harm the patients or that the patients were too ill to participate. Among the participating MHCPs, some expressed concerns during the trial regarding patients’ vulnerability and severe psychopathology and asked if it was necessary to provide the information sheet to all eligible participants. All eligible patients (N = 170) should have received the information sheet and been invited to an information meeting. It is not possible to decide if it actually occurred. Another barrier besides these concerns was the fact that the MHCPs’ main focus was the establishment of the AOTs and not research, even though the hospital was established as a university hospital.

The challenge with having professionals invite patients to the trial was overcome by attempting to encourage the MHCPs to prioritize the trial. The frequency of contact between PI and the MHCPs was increased from once a month to at least twice a month. The MHCPs were either visited, received mailing and/or were telephoned by the PI to discuss eligible participants and encourage the MHCPs to participate in the trial. The PI was available for questions and supervision during the entire trial, both in relation to the trial procedures and the intervention. Further it was decided to accept non-participation from the 16% of the MHCPs that either denied or avoided to recruit eligible participants.

### Patients’ readiness to take part in GSD and the trial

When distinguishing recruitment challenges not related to patients from those related to patients’ readiness to take part in the trial, it became clear that the latter challenges were only a few.

Looking at the total numbers from the six teams, there were 170 eligible participants; 70% (n = 120) participated in the information meeting and 84% (n = 101) signed informed consent. In total 59% of the eligible participants signed the informed consent. In general, the eligible participants participating in the information meeting were open-minded towards research and participation in the trial. Many spontaneously expressed their motivation to participate in the trial and “helping myself and others” was the most frequently mentioned reason. The two main reasons for eligible participants to decline participation appeared to be severe psychopathology, and delusional thinking related to the trial, for example that personal data would be shared with the FBI or the Government. Reasons for declining to participate in the information meeting were severe psychopathology, timing of approach and practical barriers. However, those reasons were expressed by the MHCPs and not by the eligible participants, as the PI was not in contact with the eligible participants until the information meeting.

No new strategies were employed to meet challenges with eligible participants, as the eligible participants demonstrated high readiness to take part in the trial.

Further withdrawal from treatment and dropout from the trial were calculated. In total 7% of the participants withdrew from the treatment in the trial; 3% never started working with the GSD method (2/3 of these participants stayed in the trial with data collected at all times), and during the trial 4% stopped treatment before it was completed. The reasons reported for withdrawal from treatment were; timing of approach (n = 3), death (n = 1), moving out of the country (n = 1), pregnancy (n = 1) and loss of a family member (n = 1).

The total dropout rate was 8% of the participants (3% in the control group and 5% in the intervention group). As some participants both withdrew from treatment and dropped out of the trial, the reasons reported for dropout were similar with the reasons reported for withdrawal: timing of approach, moving out of the country, loss of a family member, severe psychopathology and a brain injury.

## Discussion

Addressing challenges and barriers to recruitment encountered in this trial appeared to be fruitful and led to the conclusion that barriers, not related to patients, were at play in contrast to what is often reported by a great majority of the research literature. Through overcoming these challenges, we actually identified remarkably high patient readiness to take part and a low dropout rate.

Three types of recruitment challenges not related to patients were identified and met during the trial: 1) organizational challenges, 2) challenges with finding eligible participants and 3) challenges with having professionals invite patients. The challenges were overcome through: 1) extension of time, 2) expansion of the clinical recruitment area and 3) encouragement of professionals to invite patients to the study.

### Challenges to recruitment not related to patients and strategies to overcome them

To our knowledge organizational challenges have not prior been described as recruitment barriers. Our trial was planned according to the original implementation plan for the AOTs. As a consequence of the unanticipated changed plan, we first experienced a delay in the implementation, which was met with a time extension, a commonly used strategy in trials [27]. As mentioned by Halpern et al. [10], delays lead to increased costs, which also was the case in our trial, meaning that we had to apply for more external funding and ask the hospital management to ensure financial support. Extension of time in itself is rarely adequate to achieve the original recruitment target [27].

The next challenge that occurred due to the changed implementation plan was finding eligible participants, as the total number of eligible participants was reduced to 66% in the AOTs. Consequently we had to apply an additional strategy: expanding the clinical area. We knew that some of the patients in the DTs were eligible to receive treatment in an AOT, since an assessment of all outpatients conducted ahead of the implementation had revealed existence of patients fitting the criteria, although the AOTs had insufficient capacity for including them.

Organizational changes can happen during a trial and affect the design, so the consequences can be early termination or adaptation of the trial design. We adapted the trial design without crucial alterations, as the randomized controlled trial design is a very rigorous design.

The third barrier not related to patients was challenges having professionals invite eligible participants to the trial.

The MHCPs were the primary access to the eligible participants and the ones conducting the intervention. Primary MHCPs can play an important role in facilitating the recruitment process, as most patients trust them [19].

Some of the MHCPs were not willing to or hesitant to inform or recruit eligible participants. They expressed concern about patients’ capability to participate in research or make decisions about participation in research, and believed that the trial might complicate patients’ psychopathology. The MHCPs’ lack of knowledge of research and research methods is known to be a barrier [2,11,28]. The MHCPs in the trial were mostly registered nurses (n = 33) without much knowledge of or prior experience with research. Nursing Science in Denmark has a very short tradition for conducting research in clinical practice. To overcome the lack of knowledge, we beforehand provided the MHCPs with information about the trial, the recruitment process, and training in utilizing the GSD method. During the trial the MHCPs were provided with frequent contacts, which originally was decided to be once a month, but increased to be twice a month.

Still some of the MHCPs seemed to assess eligibility according to their own inclusion criteria, assumedly because of a personal idiosyncratic belief that patients need protection against research, which has been described by Howard et al. [2]. MHCPs are generally found lacking confidence in patients’ ability to make decisions [29], though patients are both willing and able to be involved in decisions [30]. Especially decisional capacity regarding participation in research is of great concern to MHCPs, but the presence of a diagnosis of schizophrenia is not an indication that an individual should be unable to give competent consent to research participation [13]. Of note, the ability of individuals with schizophrenia to make competent decisions relates more to their overall cognitive functioning than to the severity of specific positive or negative symptoms [31-33]. In addition, research points towards the fact that individuals with schizophrenia, who reported more severe psychiatric symptoms, expressed less willingness to participate in research [14]. The latter is in accordance with the reasons given for refusal to participate by some of the eligible participants in our trial and indicates that individuals with severe psychopathology are able to take care of themselves, when asked to participate in research.

We did not ask the MHCPs structured questions about their attitudes of recruiting participants to the trial. However, most of the MHCPs expressed their opinions through spontaneous conversation, comments or even avoidance, and it did not differ from what has been described in prior studies [2,11].

### Patients’ readiness to take part in GSD and the trial

After the recruitment challenges not related to patients were overcome, we identified remarkably high patient readiness to participate in the trial, a low withdrawal from treatment and a low dropout from the trial.

In general, the eligible participants were open-minded and positive towards the trial when they participated in the information meeting. We cannot conclude that all eligible participants were positive towards the trial, as the PI only spoke to the eligible participants who participated in the information meeting. In total 59% of the total eligible participants signed informed consent, but 84% of the eligible participants participating in the information meeting signed informed consent. Those numbers are remarkably high comparing to the study of Zullino et al. [19], who reported an overall hypothetical acceptance rate above 70% among in-patients with severe mental illnesses, with a general lower acceptance rate among individuals with schizophrenia, expecting this number to be much lower in a real recruitment situation.

The reasons expressed by the eligible participants that declined to participate in the trial were severe psychopathology or delusions. Those reasons are in agreement with those found in the literature [16,17,34]. We did not ask the participants directly for their motivation to participate, but those who expressed it spontaneously often stressed “helping other people or helping myself” as an important factor to participate in the trial, which were in accordance with the findings from Chong et al. [15] and Roberts et al. [18].

Both the rate of withdrawal from treatment (7%) and total dropout from our trial (8%) were lower than reported in reviews [5,9]. Villeneuve et al. [9] reported a withdrawal of 13% from their review. Our trial lasted for 52 weeks and the withdrawal number from the review was reported for trials lasting approximately 26 weeks on average. Additionally, Villeneuve et al. [9] reported factors that might have a negative effect on the withdrawal rate such as, increased age, longer illness duration, longer treatment duration and being male. Our participants were out-patients with a mean age of 37.5, illness duration above 10 years, treatment duration with the GSD method was 26 weeks and 47% of the participants were men. It seems that the factors mentioned in the review by Villeneuve et al. [9] did not apply fully to our study indicating that other factors might be at play regarding impact on withdrawal rates. Additionally, they also reported factors that might have a positive effect on the withdrawal rate as, study quality and hospitalized subjects [9]. The participants in our trial might have found the GSD intervention relevant and useful, and the study quality high.

Commonly, dropout rates in trials are reported to be high. According to Gilbody et al. [5] in trials of new antipsychotics, up to 60% dropped out, and in a meta-analysis [8] of trials assessing antipsychotic medication in the treatment of schizophrenia, dropout rates ranged from 0-65.9%, showing that the duration of the trial increased dropout rates remarkably.

Gilbody et al. [5] suggested that minimal effort on the part of participants and minimal deviation from routine care, and understanding what the patients perceive as a relevant outcome, might have an impact on the motivation of patients with schizophrenia to participate and stay in trials. Taking Gilbody’s suggestions into account, this trial was an effectiveness trial within a real world setting. Participants were seen in their own homes during the intervention, but also during the data collection. As mentioned prior, the participants might have found the GSD intervention relevant and useful, but also the outcome measures of the trial might have been relevant and motivated the participants to participate and stay in the trial.

## Conclusion

Three types of recruitment challenges not related to patients were identified and overcome during the trial. By overcoming these challenges we found remarkably high patient readiness to take part and to complete the trial. The distinction between the recruitment challenges was important in discovering the readiness among patients with schizophrenia to take part in and complete a trial with the GSD intervention.

The limitations of this study are that the challenges and the strategies are descriptive, based on data, notes and observations from the trial. We cannot directly confirm that the strategies caused the completion of the trial; we can only assume that the strategies had an impact. Moreover we have not further investigated whether or how the patients’ readiness to take part and complete the trial was connected with a perception of high quality of the GSD intervention as expressed by one patient in a case study. Especially the patient-provider collaboration and the content of the reflection sheets were emphasized as very important contribution to feeling believed and in charge [26].

The strength of the study is that we openly report recruitment challenges and strategies to provide methodological transparency of this trial to other researchers. Further, we address organizational challenges and barriers, which have been given limited attention until now, but seem to be important factors that researchers need to consider when planning or revising future trials.

Most importantly, we have drawn attention towards the value of thoroughly distinguishing between recruitment challenges. If we do not question the assumption that recruitment challenges are mostly related to the patients, researchers might overlook the importance of and ability to overcome other recruitment challenges. By overcoming them we might identify which interventions patients with schizophrenia prefer to take part in which can provide information for further investigating what the patients find beneficial to assist them in more effectively living with their illness.

## Abbreviations

AOT: Assertive outreach team; DT: District team; RCT: Randomized controlled trial; GSD: Guided self-determination; MHCP: Mental health care professional; PI: Principal investigator.

## Competing interests

The authors declare that they have no competing interests.

The study was funded by Aalborg Psychiatric Hospital, The Novo Nordisk Foundation, The Health Insurance Foundation, and TrygFonden, but they had no influence on planning the study or preparing the manuscript.

## Authors’ contributions

Authors RJ, PMJ and VZ designed the study and wrote the protocol. Author RJ managed the literature searches and the data collection. Authors RJ and VZ wrote the first draft of the manuscript. Authors PMJ, KB, PL and LH commented on the first manuscript and contributed to the writing of the subsequent manuscript versions. All authors contributed in the finalization of the manuscript and have approved the final version.

## Pre-publication history

The pre-publication history for this paper can be accessed here:

http://www.biomedcentral.com/1471-244X/14/28/prepub
